# Fast interfacial charge transfer in α-Fe_2_O_3−δ_C_δ_/FeVO_4−x+δ_C_x−δ_@C bulk heterojunctions with controllable phase content

**DOI:** 10.1038/srep38603

**Published:** 2016-12-07

**Authors:** Chengcheng Zhao, Guoqiang Tan, Wei Yang, Chi Xu, Ting Liu, Yuning Su, Huijun Ren, Ao Xia

**Affiliations:** 1 School of Materials Science and Engineering, Shaanxi University of Science & Technology, Xi’an 710021, China

## Abstract

The novelties in this paper are embodied in the fast interfacial charge transfer in α-Fe_2_O_3−δ_C_δ_/FeVO_4−x+δ_C_x−δ_@C bulk heterojunctions with controllable phase compositions. The carbon source-glucose plays an important role as the connecting bridge between the micelles in the solution, forming interfacial C-O, C-O-Fe and O-Fe-C bonds through dehydration and polymerization reactions. Then the extra VO_3_^−^ around the FeVO_4_ colloidal particles can react with unstable Fe(OH)_3_, resulting the phase transformation from α-Fe_2_O_3_ (47.99–7.16%) into FeVO_4_ (52.01–92.84%), promoting photocarriers’ generation capacities. After final carbonization, a part of C atoms enter into lattices of α-Fe_2_O_3_ and FeVO_4_, forming impurity levels and oxygen vacancies to increase effective light absorptions. Another part of C sources turn into interfacial carbon layers to bring fast charge transfer by decreasing the charge transition resistance (from 53.15 kΩ into 8.29 kΩ) and the surface recombination rate (from 64.07% into 7.59%). The results show that the bulk heterojunction with 90.29% FeVO_4_ and 9.71% α-Fe_2_O_3_ shows ideal light absorption, carriers’ transfer efficiency and available photocatalytic property. In general, the synergistic effect of optimized heterojunction structure, carbon replacing and the interface carbon layers are critical to develop great potential in stable and recoverable use.

The utilization of solar energy makes photocatalytic materials an important alternative to solving the present energy source and environmental pollution crises. However, there are two important problems limit their practical applications: the poor energy conversion efficiency is faced by almost all kinds of the photocatalysts^1^; the incomplete mineralize of dyes, coupled with the difficulty in recycling using of photocatalysts, makes it difficult to avoid secondary pollution. To solve these problems, the explorations on various photocatalysts with magnetic recoverable potential and the modifications on absorption-transition-action processes are in constant development and advancement.

As we know, hematite (α-Fe_2_O_3_) is an n-type semiconductor (Eg = ca.2.1 eV) with the space group of R-3C^2^,^3^,^4^, which is one promising candidate for solving the secondary pollution and extending the photocatalytic adaptability. But it can only work effectively in the Fenton system. In order to increase accessible practical light response and carriers’ transfer efficiency, a narrow-gap semiconductor is needed to establish the recommended heterojunction structure^5^,^6^,^7^,^8^. Among many alternative compounds, triclinic FeVO_4_ is a stable phase (Eg = ca.2.05 eV) at room temperature with its magnetic, electrical and photocatalytic properties being observed^9^,^10^,^11^. Therefore, the heterojunctions consisting of α-Fe_2_O_3_ and FeVO_4_ will have great study potential^12^, especially being prepared from simple raw materials. In addition, better than the common heterojunctions, the bulk heterojunctions have interfaces almost everywhere within the samples. Photocarriers can be transferred to the interfaces through short paths, improving the carriers’ separation efficiency^13^,^14^,^15^.

Currently, integrating two or more modification means is the developing trend on improving the photocatalytic properties, such as doping ions or atoms in the hetrojunctions^16^,^17^. As is known to all, there is a hard gradient gap between the surface active sites and bulk centers for the photocatalytic reaction driving forces to transfer. Actually, the interfacial activities and correlations play as the key role to decide the kinetics and degrees of the whole reactions. Therefore, developing meaningful interfaces is important. Among many candidates, C doping is of great particularities to accomplish nonmetal replacement and create carbon or carbon containing interfaces^18^,^19^, which can be achieved by C-containing additives, such as the common glucose^20^,^21^. According to the present reports, it can be made sure that the π-π interactions within the carbon networks between the catalysts are beneficial to transfer electrons. On the basis of the above interface effects, the surface light absorption, the interface charge transfer, the surface molecules adsorption and the interfacial redox reaction will have an obvious enhancement^22^.

In this work, the heterojunction structure and the C doping are used to improve the photocatalytic activity. The phase content evolution in the heterojunction and the interface effect on electrons transfer are mainly studied. For the further detailed information, a series of novel electrochemical measurements are employed to help to analyze the light response, carriers’ separation and charge transfer in the reaction system.

## Results and Discussion

**Figure 1:**
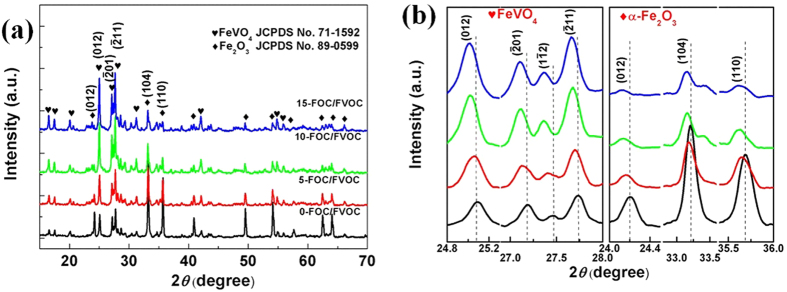
(**a**) XRD patterns of FOC/FVOC heterojunctions, (**b**) respectively magnified diffraction peaks of FeVO_4_ and α-Fe_2_O_3_ at 24–36°.

In this article, the C-doped heterojunctions consisting of FeVO_4_ and α-Fe_2_O_3_ were prepared and studied. In Fig. 1a, the peaks can be indexed into α-Fe_2_O_3_ (JCPDS No. 89-0599) and FeVO_4_ (JCPDS No. 71-1592). With the C content increasing, there are obvious left shifts in main peaks of both α-Fe_2_O_3_ and FeVO_4_ by about 0.08° and 0.06° respectively (Fig. 1b) due to the increased interplanar spacing in both lattices of FeVO_4_ and α-Fe_2_O_3_. Because the C atoms (0.077 nm) are approximately similar to the O atoms (0.074 nm), it suggests that the C atoms can replace the lattice O atoms in FeVO_4_ and α-Fe_2_O_3_, resulting in lattice distortions. Moreover, the intensities of the (012), (−201) (1–12) and (−211) peaks of FeVO_4_ are increasing while those of α-Fe_2_O_3_ like (104) and (110) are decreasing (Fig. 1b). For a more intuitive comparison, the ratios of most intense diffraction peaks (I_FVOC(−211)_/I_FOC(104)_) of the samples are calculated, as listed in Table 1. Form 0-FOC/FVOC to 15-FOC/FVOC, the I_FVOC(−211)_/I_FOC(104)_ value increases from 0.362 to 1.268, suggesting the increased relative content of FeVO_4_ in the heterojunctions^23^.

In order to further define the above trends, the Rietveld refinements were performed on the base of XRD data using MAUD and FindIt softwares within a reasonable error limit (R-factor < 15%), as shown in the supplementary Fig. S1 and the Table 1. The FeVO_4_ (ICSD: 10329) adopts triclinic P-1 structure and the α-Fe_2_O_3_ (ICSD: 15840) adopts trigonal R-3c:H structure. With the C content increasing from 0% to 15%, the content of FeVO_4_ increases from 52.01% to 92.84% while that of α-Fe_2_O_3_ decreases from 47.99% to 7.16%, which is in accordance with above increased I_FVOC(−211)_/I_FOC(104)_ values. Therefore, it can be made sure that during the C atoms entering into the lattices, the development of triclinic FeVO_4_ rather than trigonal α-Fe_2_O_3_ is promoted. In other words, the phase compositions in the heterojunctions FOC/FVOC are controllable.

The morphology evolutions along with increasing glucose additive were observed by SEM and TEM in supplementary Fig. S2. In Fig. S2a,b, the 0-FOC/FVOC behaves one kind of smooth bulks morphology with two kinds of phases, suggesting the FOC/FVOC bulk heterojunctions. The high energetic facets of FeVO_4_ and α-Fe_2_O_3_ have high atomic stacking rates, leading to kinetics variations in different facets, exposing them more on respective (012) sides with the lattice spacing of 0.3595 nm and 0.3738 nm^24^ (Fig. S2c). With C content gradually added, the bulks grow into plate-like crystals and get closer to connect with each other. What’s more, as further observed in 10-FOC/FVOC (Fig. S2e–j), the plates’ surfaces are surrounded by carbon layers with the thickness of 0.7–0.9 nm which is equal to the thickness of 10–13 C atoms. The exact C element EDS mapping is also detected (Fig. S2k). Obviously, the glucose additive makes sense to crystal growth as well as forming average surface carbon layer coverage.

**Figure 2:**
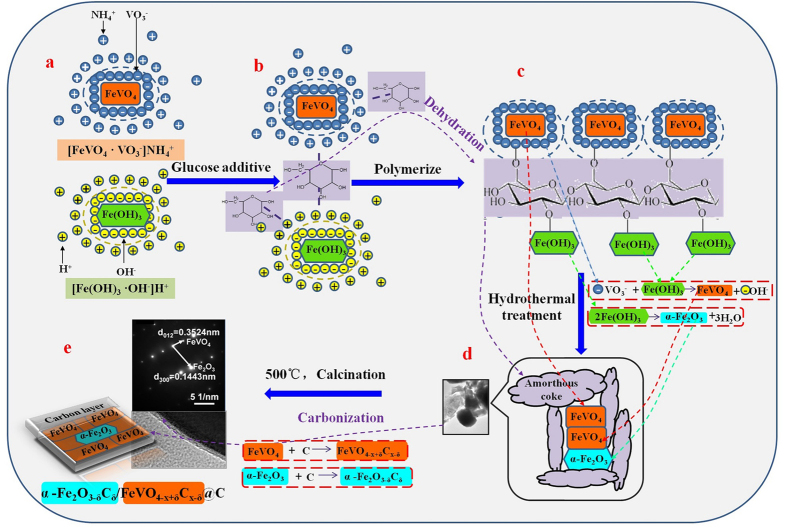
Phase transformation mechanism and sample evolution processes.

According to the above analyses, it can be made sure that the crystal transition and growth can be controlled by the glucose additive. In order to further study the controlling ways and processes, the zeta potentials (ζ values) of samples are combined with the phase content in supplementary Fig. S3. With the glucose content increasing from 0 to 15%, the suspensions’ negative ζ values decreases from −33.8 mV to −12.7 mV companied with the phase transformation. It suggests certain correlations between the glucose additive and the crystal evolution processes, for which the mechanism is proposed as shown in Fig. 2 and following equations 1, 2, 3, 4, 5, 6, 7, 8, 9, 10, 11.

First, after the raw materials of FeCl_3_·H_2_O and NH_4_VO_3_ being dissolved in the water, the FeVO_4_ and Fe(OH)_3_ crystal seeds are produced first, as shown in equations 1, 2, 3, 4, 5 25,26. In the precursors, the FeVO_4_ colloidal particles with extra VO_3_^−^ ions distributing around can absorb the oppositely charged NH_4_^+^ ions, forming the [FeVO_4_·VO_3_^−^]NH_4_^+^ micelles (equation 6, Fig. 2a). Similarly, the [Fe(OH)_3_·OH^−^]H^+^ micelles are formed (equation 7, Fig. 2a). Before the glucose added, the blank 0-FOC/FVOC is negatively charged with ζ value of −33.8 mV. After the participation of glucose molecules, because of the dehydration polymerizations among OH^−^ ions on the edges of the C_6_H_12_O_6_ molecules, positioning OH^−^ ions of the Fe(OH)_3_ colloidal particles and OH^−^ ions bonded by the NH_4_^+^ ions^27^, the dispersive micelles are integrated into [FeVO_4_·VO_3_^−^]-CH_2_O(CHOH)_4_CO-[Fe(OH)_3_·OH^−^] groups^28^ (eqution 8, Fig. 2b). The formed C-O- bonds have stronger affinity for nucleus than OH- so that the Fe(OH)_3_ colloidal particles can effectively contact the [FeVO_4_·VO_3_^−^] colloidal particles (equation 9, Fig. 2c). Then under the hydrothermal treatment, VO_3_^−^ ions react with the active Fe(OH)_3_ colloidal particles, resulting in the critical crystal transformation from Fe(OH)_3_ into FeVO_4_ (equation 10, Fig. 2d). The residual Fe(OH)_3_ dehydrate into the final α-Fe_2_O_3_. With the content of glucose increasing, the decrease of ζ value into −12.3 mV can weaken the repulsive forces, suggesting that the micelle-binding between the Fe(OH)_3_ and [FeVO_4_·VO_3_^−^] are effectively promoted, promoting more phase transformation as well as urging the bulks to grow larger and closer^29^, which is in accordance with the morphology evolution discussed above. Such transformation within the micelle groups results in the final production of bulk α-Fe_2_O_3_/FeVO_4_ heterojunctions which have short transferring paths for photocarriers. At the same time of the phase transformation during the hydrothermal treatment, the glucose molecules polymerize into oligosaccharides first and then into amorphous cokes, wrapping up the α-Fe_2_O_3_/FeVO_4_ cores. After 500 °C calcinations (equation 11, Fig. 2e), a part of the amorphous cokes carbonize into carbon layers wrapping around the bulk heterojunctions. Another part of C atoms can enter into the lattices of α-Fe_2_O_3_ and FeVO_4_ to replace O and leave O_vac_, forming the final α-Fe_2_O_3−δ_C_δ_/FeVO_4−x+δ_C_x−δ_@C samples.

In general, the glucose molecules have strong and delicate correlations between each other. They play an important role as a connecting bridge to help the VO_3_^−^ seize Fe^3+^ from Fe(OH)_3_, resulting in the phase transformation from α-Fe_2_O_3_ into FeVO_4_. The more glucose is added, the stronger connecting effect will be. Therefore, from 0-FOC/FVOC to 15-FOC/FVOC, it comes up with the suppressed growth of α-Fe_2_O_3_ from 47.99% to 7.16% and the promoted growth FeVO_4_ from 52.01% to 92.84%. And also, the α-Fe_2_O_3_/FeVO_4_ heterojunctions are wrapped by external carbon layers and invaded by internal entered carbon atoms in both phases, forming the target α-Fe_2_O_3−δ_C_δ_/FeVO_4−x+δ_C_x−δ_@C samples. Obviously, the interfaces among the heterojunctions will be greatly important.


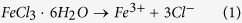



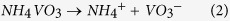



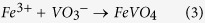



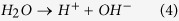



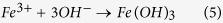



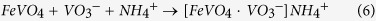



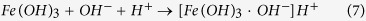



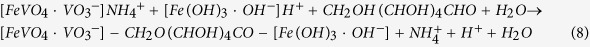



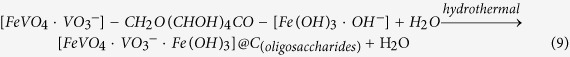



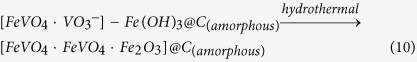



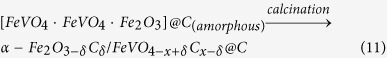


**Figure 3:**
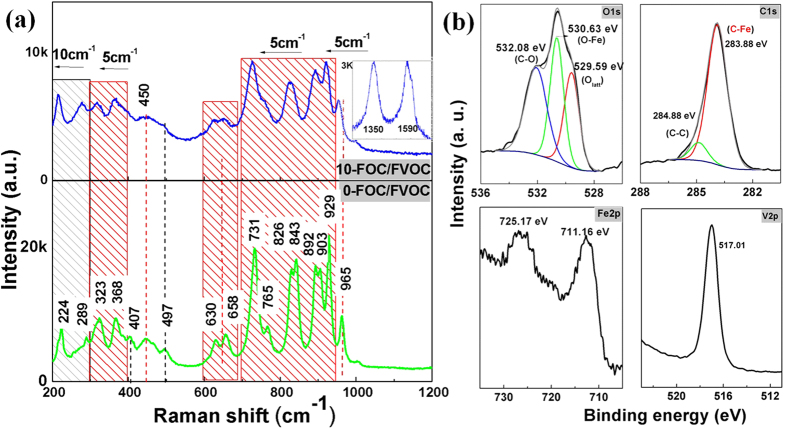
(**a**) Raman spectra of 0-FOC/FVOC and 10-FOC/FVOC, and (**b**) XPS of O1s, C1s, Fe2p and V2p of 10-FOC/FVOC.

In order to define the above interfacial characteristics, the Raman spectra and XPS spectra of samples were measured in Fig. 3. In Fig. 3a, the α-Fe_2_O_3_ contain peaks (black masks) at 224 cm^−1^, 289 cm^−1^ and 407 cm^−1^ 26. The FeVO_4_ contain peaks (red masks) at 323 cm^−1^, 368 cm^−1^, 630 cm^−1^, 658 cm^−1^, 700–950 cm^−1^ and 965 cm^−1^ 30,31,32,33,34. The typical G band of the first order scattering *sp*^*2*^ domains and D band of *sp*^*3*^ defects^35^ are shown in the inset of Fig. 3a. Remarkably, the overall blue shift of 5–10 cm^−1^ due to the increased bond lengths, the mergers of split peaks (892 cm^−1^ and 903 cm^−1^) corresponded to gradually consistent V-O bonds and the slacks of small peaks at 630 cm^−1^ and 658 cm^−1^ related to decreased V-O-Fe bridges’ vibrations^35^ can help to define that the added C can leave structural distortions, break initial O containing bonds and form new bonds^36^,^37^.

In order to confirm such new bonds, the surface chemical compositions and states of 10-FOC/FVOC were further determined by the XPS in Fig. 3b. The Fe2p and V2p spectra suggest no chemical state change in Fe^3+^ and V^5+^. The new bonds are mainly demonstrated by O1s and C1s spectra. The O1s spectrum contains three peaks. The peak at 529.59 eV is due to lattice oxygen (O_latt_)^38^. The peaks at 532.08 eV and 530.63 eV are attributed to C-O and Fe-O-C derived from surface absorbed oxygen (O_ads_)^39^,^40^. The C1s peak can be deconvoluted into two peaks: one at 284.88 eV of the C-C bond and the other more important and stronger one at 283.88 eV of Fe-C or O-Fe-C bond^41^,^42^. The formation of Fe-C or O-Fe-C can directly further confirm the C atoms replacement for O atoms, which will leave active O_vac_^43^. Therefore, it can be made sure that added glucose after carbonization can form interfacial C-O and Fe-O-C bonds during the crystal transformation and growth, setting up effective bond correlations between the surface carbon layer and heterojunctions. At the same time, the C atoms can replace O atoms to form O-Fe-C bonds and leave O_vac_ for trapping electrons to form active groups for photocatalytic reactions.

**Figure 4:**
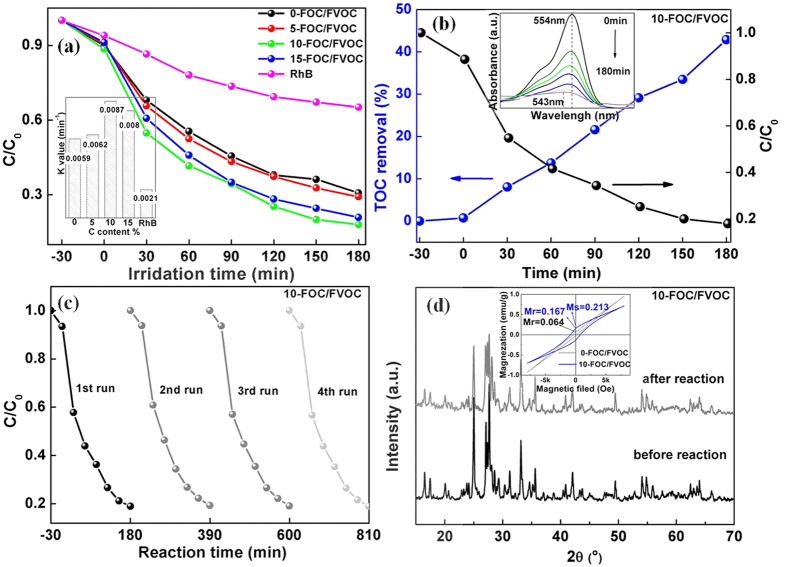
(**a**) Photocatalytic degradation rates of RhB (The inset shows the corresponding reaction rate constants of FOC/FVOC heterojunctions), (**b**) comparison of TOC removals and RhB degradation rates of 10-FOC/FVOC, (**c**) recycle experiments of 10-FOC/FVOC and (**d**) XRD patterns of 10-FOC/FVOC before and after the recycle experiments (The inset shows the magnetic hysteresis loops of 0-FOC/FVOC and 10-FOC/FVOC at room temperature).

To measure the photocatalytic efficiency, the RhB concentration variations during the decolorization by FOC/FVOC heterojunctions were recorded and calculated as shown in Fig. 4a. Their dynamic constants (K values) were fitted and shown in the inset of Fig. 4a. All the FOC/FVOC heterojunctions show significant photocatalytic activities under the UV light, of which the 10-FOC/FVOC has the highest degradation rate of 83% within 180 min with K = 0.0087 min^−1^. The total organic carbon (TOC) removals of 10-FOC/FVOC were also detected to evaluate the mineralization capacity, as plotted in Fig. 4b. The TOC removal of 10-FOC/FVOC is 43%, which is naturally lower than the degradation rate due to the incomplete degradation from RhB molecules into CO_2_ and H_2_O. The incomplete degradation results in the blue shift of RhB’s absorption peaks from 554 nm to 543 nm, corresponding to RhB and N,N,N0-triethylated Rhodamine (TER) (inset of Fig. 4b). Except for the effective photocatalytic activities, these photocarriers also contain good stability (Fig. 4c,d). The photocatalytic behavior of 10-FOC/FVOC can maintain at least four runs in a cycle experiment with a little activity loss and a stable structure. Moreover, the C doped FOC/FVOC heterojunctions have improved magnetic properties (Ms = 0.213 emu/g), suggesting their magnetic recovery potential (inset of Fig. 4d).

So, here comes the question: why 10-FOC/FVOC has much higher photocatalytic properties? Probably, the keys lie in the improved light absorption ability, effective charge transfer process within and among the heterojunctions. In order to prove it, the electrochemical measurements were mainly employed in this section.

**Figure 5:**
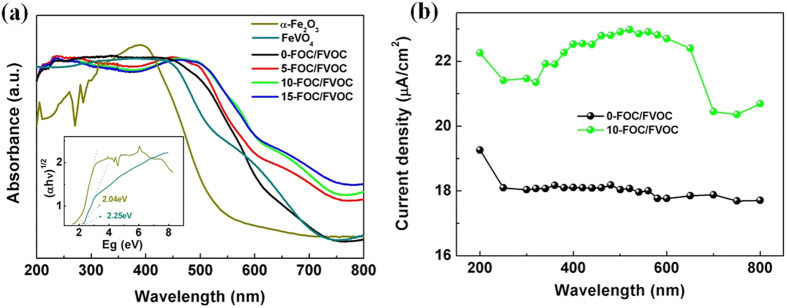
(**a**) DRS spectra of FOC/FVOC and pure phases and (**b**) photocurrent action spectra of 0-FOC/FVOC and 10-FOC/FVOC.

First of all, the DRS spectra of FOC/FVOC and pure phases were shown in Fig. 5a. The band gaps of α-Fe_2_O_3_ and FeVO_4_ are fitted in the inset image to be 2.25 eV and 2.04 eV, respectively. The heterojunctions have two linear absorptions at 500–600 nm and 600–700 nm which are corresponded to the phase composition and the C1s impurity level^44^ respectively. With the increase of doping C, the obvious overall increased absorption and red-shift can be found which is attributed to two reasons: the increase of narrower gap FeVO_4_’s content in the heterojunctions and the enhancement of C1s level.

In order to study its actual efficiency in producing photocarriers, the photocurrent densities of 0-FOC/FVOC and 10-FOC/FVOC under monochromatic light at 200–800 nm are recorded in Fig. 5b, Interestingly, the broad peak in the action spectrum of 10-FOC/FVOC film is consistent with its DRS spectrum, showing much higher current density than 0-FOC/FVOC, suggesting that doping C can effectively improve the carriers’ transfer efficiency. In other words, the important meaning of C doping for improving the light absorption is to improve the effective transfer of active carriers rather than only to improve the surface light response.

**Figure 6:**
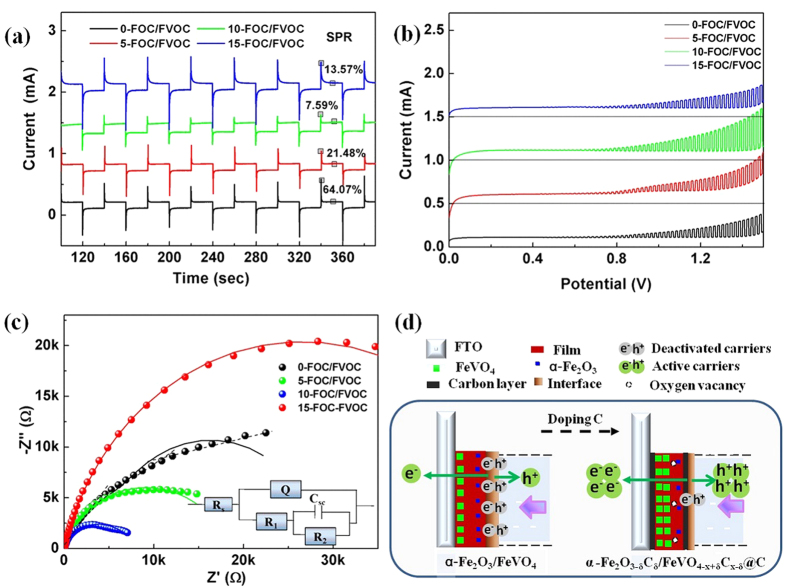
(**a**) Current vs. Time curves, (**b**) Current vs. Time curves, (**c**) EIS spectra and (**d**) charge transfer mechanism of FOC/FVOC film electrodes.

For an exploration of charge transfer efficiency, chopped light chronoamperometry currents were measured. In Fig. 6a, it shows the Current vs. Time curves with a chopped time of 20 s. The transient current can reach a stable state after a peak value at the moment of light on or light off. That means there is a transfer delay of electrons due to the electrode/electrolyte interface barriers, resulting in directly surface recombination of carriers. In order to evaluate the degree of such surface recombination, the peak currents at 340 s are recorded as I_max_ and the transient currents at 350 s are recorded as I_ss_ to calculate the surface photocarriers recombination rates (SPR) according to the equation 12 45,46. The SPR of 10-FOC/FVOC is the lowest 7.59% which has a very big decrease from the initial 0-FOC/FVOC of 64.07%. That means the surface recombination can be effectively relieved to improve the charge transfer efficiency, generating strengthened photocurrent.


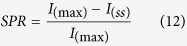


In Fig. 6b the Current vs. potential curves, it can be seen that the FOC/FVOC film electrodes have effective transient currents in a very short response time of 2 s. The open-circuit voltage at zero current (Voc) is about 0.6 V. It is obvious that after C doping, the intensities of the currents are improved compared to that of blank heterojunctions. The 10-FOC/FVOC has the highest current at the same potential of 1.5 V, suggesting its highest rising rate. Generally, the rising rate corresponds to the inverse contact resistance (R_c_) of the electrode/electrolyte interface^47^,^48^. So it can be deduced that the C doped FOC/FVOC have the decreased R_c_ values of the electrode/electrolyte interfaces to favor the fast charge transfer, supporting the improved charge transfer efficiencies.

For a clear understanding of such fast charge transfer among the heterojunctions, the electrochemical impedance spectroscopies (EIS) were shown via the EIS Nyquist plots (Fig. 6c). The used circuit model was R(Q(R(CR))), where Rs was solution resistance, Q was electrochemical double-layer capacitance, R_1_ was electrolytic resistance, C_sc_ was space charge capacitance and R_2_ was charge transfer resistance (R_ct_). The fitting data were listed in supplementary Table S2. The curve of 0-FOC/FVOC is nearly linear, suggesting the ions diffusion is the speed deciding process. The C-doped FOC/FVOC hetrojunctions curves are smooth arcs, suggesting the charge transfer is the speed deciding process. As we know, in a typical Nyquist plot, the response order is pure resistance, then capacitance and electrochemical process, and then diffusion process^49^,^50^. So, the respond sensibility of 0-FOC/FVOC is higher than heterojunctions with C. It looks like that the doping C delays the response of electrodes. But in above results, the currents with C doping are higher. In this view, the actual carrier density is considered to be the key factor. Without C doping, the light absorption and the current density are lower, the actual completed charge transfer is much lower with serious carriers’ recombination. So the responses time of resistance, capacitance and electrochemical processes are short. After C doping, similarly, the much higher current density needs longer charge transfer process, besides that the C within and among the hetrojunction are obstacles to ions diffusion to some degree even though they can help the charge transfer. It is easy to make a judge and choose that the actually effective carrier amount is the criteria for judging the photoctalytic properties. In addition, the radius of the arc on the Nyquist plot reflects the value of reaction resistance (R_ct_/2) and the separation efficiency of electron-hole pairs^51^. As shown in supplementary Table S2, the Rct value has an obvious decrease with C doping, and the 10-FOC/FVOC has the minimum resistance of 8.29 kΩ. Therefore, it can be made sure that the C doped heterojunctions have decreased transfer barriers among the heterojunctions due to the surface carbon layers.

On the basis of the above analysis, the electron transition mechanism of the FOC/FVOC electrodes was illustrated in Fig. 6d. The different colors represent different meanings, as listed above the film electrodes. Before doping C, FeVO_4_ and α-Fe_2_O_3_ exist within the electrodes. When the film side is exerted illumination, most of the carriers are generated near the electrode/electrolyte interfaces, on which the serious combination and deactivation of carriers happens. Finally, only a small amount of electrons can go across the film toward FTO to form the photocurrent^52^. After doping C, the phase transformation and the carbon layer on the film surface can be seen, which have much lower interface barriers and charge transfer resistances. The fast charge transfer across the carbon layer interfaces help to remove the transfer delay, release the surface recombination and allow more electrons with higher densities to pass. In such a way, the photocarriers’ separation and transportation efficiencies have been improved mainly due to the interfacial fast transfer.

**Figure 7:**
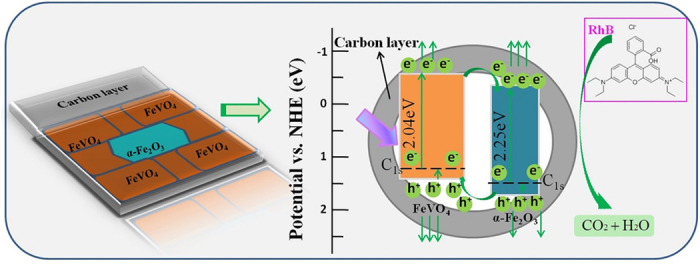
The photocatalytic degradation mechanism of FOC/FVOC heterojunctions.

According the above analyses, the proposed photocatalytic degradation mechanism with doping C was shown in Fig. 7. The improved light absorption and charge transfer efficiency are the reasons for the improvement of photocatalytic properties. Specially speaking, by doping C, the controllable phase transformation makes the heterojunctions contain more FeVO_4_ with narrower gap, forming C1s levels in both phases, resulting in wholly decreased generation gap and improved light absorption to generate more photocarriers. In the heterojunction structure, the electrons (e^−^) of FeVO_4_ can be transferred across the space charge region to the conduction band of α-Fe_2_O_3_ as well as directly diffusing to the surface, similarly to the transfer of the holes (h^+^), improving the carriers’ separation efficiencies. Then, the most important of all, the fast interface charge transfer achieved by carbon layers makes great contribution to the decrease of the interfacial transfer resistance, avoiding massive carriers’ consuming by surface recombination, and promising the constant activities of the carriers. Such carbon layers can also adsorb the RhB molecules easily. In addition, the active surface O_vac_ can easily catch the dissolved oxygen to form active groups. Then, the effective active photocarriers can be degraded by the redox reactions.

## Conclusions

In summary, the C doped α-Fe_2_O_3−δ_C_δ_/FeVO_4−x+δ_C_x−δ_ heterojunctions with important effective interfacial carbon layers are prepared in this work. The glucose acts as the phase control agent to help the VO_3_^−^ seize Fe^3+^ from Fe(OH)_3_, resulting in the suppressed growth of α-Fe_2_O_3_ from 47.99% to 7.16% and promoted growth FeVO_4_ from 52.1% to 92.84% in the bulk heterojunctions. The set-up of Fe_2_O_3_/FeVO_4_ bulk hetrojunctions can effectively separate and transfer the electron-hole pairs in short paths. After dehydration and carbonization, a part of carbon in the lattice can form impurity levels and leave oxygen vacancies to increase the absorption range. The outside of the α-Fe_2_O_3_/FeVO_4_ heterojunctions are wrapped with carbon layers, forming the final α-Fe_2_O_3−δ_C_δ_/FeVO_4−x+δ_C_x−δ_@C. Because of the fast charge transfer in the interface with the decreased barrier, greatly improved effective carriers can pass the surface to form active groups. Therefore, under the synergistic effect of increased light response, optimized heterojunction structure and effective charge transfer, the photocatalytic property is obviously improved from 69.3% to 83%. And also, the as prepared α-Fe_2_O_3−δ_C_δ_/FeVO_4−x+δ_C_x−δ_ heterojunctions have great potential in stable and magnetic recoverable use in its practical application.

## Experimental Section

### Heterojunctions preparation

All the reagents were of analytical grade and were used without any further purification in this article. Typically, 3mmoL FeCl_3_·6H_2_O and 3mmoL NH_4_VO_3_ were dissolved in 15 mL deionized water respectively. After magnetic stirring for 30 min, the two solutions were mixed together with the molar ratio of 1∶1 and adjusted by NaOH solution to pH = 8. Then, the glucoses (0%, 5%, 10% and 15%) were added, with stirring for another 30 min to get the precursors. After that the precursors were transferred into Teflon-lined stainless autoclaves to carry out the hydrothermal treatment at 220 °C for 16 h. After cooling to room temperature, the precipitates were collected and washed by deionized water and ethanol, three times for each, before being dried at 70 °C for 12 h. Finally, the precipitates were calcined at 550 °C for 2 h to get the final products. For an exact expression of coexistence of C in FeVO_4_ and α-Fe_2_O_3_, the C content entering into the α-Fe_2_O_3_ lattice was donated as δ (δ < x) and that into FeVO_4_ was x − δ. Hereafter, the samples α-Fe_2_O_3−δ_C_δ_/FeVO_4−x+δ_C_x−δ_@C were denoted as x-FOC/FVOC (x = 0, 5, 10 and 15).

### Characterization

To determine the phase composition of the samples, the powder X-ray diffraction (XRD, D/max-2200PC, Rigaku Japan, CuKa, λ = 0.15406 nm, 40 kV, 40 mA) was used, with the assistance of an energy dispersive X-ray spectrometry (EDS) for chemical elements analysis (The sample was placed on the aluminum foil). The lattice information and relative phase content were fitted by the MAUD and FindIt softwares. In order to prove the interfacial interaction and structure, the laser micro-Raman spectroscopy (Raman, Renishaw-invia, U.K.) and the X-ray photoelectron spectroscopy (XPS, XSAM800, Japan) were used. The morphologies were observed on a field emission scanning electron microscopy (FE-SEM, S4800, Japan) and a transmission electron microscopy (TEM, FEI TECNAI G2F20 S-TWIN, U.S.A.). The zeta potential was measured by a Nano Particle size and zeta potential analyzer (NAMO-ZS, Malvern, UK). The UV−vis diffuse reflectance spectra (DRS, Cary 5000, Agilent, U.S.A.) were used to study the absorption range. The magnetic properties were measured by a superconducting quantum interference magnetic measuring system (M-H, MPMS-XL-7, U.S.A.).

### Photocatalytic analyses

The photocatalytic properties were measured by degradating Rhodamine B agent (RhB) in a XPA-7 photochemical reactor (Xujiang Machine Factory, Nanjing, China) with a 300 W Hg lamp as UV light source. A total of 0.05 g photocatalyst was added into initial RhB solution (50 mL, 5ppm) each time. Before illumination, an adsorption-desorption equilibrium between photocatalysts and RhB molecules was needed to achieve by stirring in darkness for 30 min. The concentrations of RhB supernatants after centrifuged were analyzed using an UV-vis spectrophotometer (SP-756P, Shanghai optical spectrometer company, China) and a total organic carbon analyzer (TOC, Liqui TOC II, Elementar, German) in a 30 min interval to calculate the final degradation rate and mineralization rate.

### Electrochemical analyses

A standard three-electrode cell was used to perform the electrochemical measurements on an electrochemical workstation (CHI660E, China). The FOC/FVOC films were placed as working electrodes, with the platinum as a counter electrode and the saturated Ag/AgCl electrode as a reference electrode. The working electrode was prepared as follows: A total of 0.1 g powder was dissolved into the mixed solution of 1 mL anhydrous ethanol and 0.1 mL acetyl acetone. After stirring and ultrasonic dispersing, the stable suspension was repeatedly spread out on a FTO glass substrate (1.5 × 2 cm^2^) for 3 times. Then the substrate was calcined at 600 °C for 2 h to get the film working electrode. The photocurrents were exhibited as Current vs. Time curves with different chopped times and Current vs. Potential curves (Electrolyte, 0.1 mol·L^−1^ Na_2_SO_4_ solution; sweep rate, 5 mV·s^−1^; light source, a 300 W Xe-lamp with a cutoff filter to achieve UV lights (λ < 420 nm) irradiation; bias voltage, 0.6 V). The EIS was obtained in the frequency range of 0.01–100000 Hz and then fitted by a software named ZsimpWin. A specified 300 W Xe lamp (HSX-F/UV, China) and a monochromator (Ommo 301, China) were employed for photocurrent action spectra.

## Additional Information

**How to cite this article**: Zhao, C. *et al*. Fast interfacial charge transfer in a-Fe_2_O_3-δ_C_δ_/FeVO_4-x+δ_C_x-δ_@C bulk heterojunctions with controllable phase content. *Sci. Rep.* **6**, 38603; doi: 10.1038/srep38603 (2016).

**Publisher's note:** Springer Nature remains neutral with regard to jurisdictional claims in published maps and institutional affiliations.

## Supplementary Material

Supplementary Information

## Figures and Tables

**Table 1:** Crystal structure information of heterojunctions based on XRD and Rietveld Refinement.

C content (%)	Phase	Weight percentage (%)	Crystal structure	Lattice parameters			R-factors (%)	I_FVOC(−211)_/I_FOC(104)_
				a(Å)	b(Å)	c(Å)		
0	FeVO_4_	52.01	Triclinic,P-1	6.7119	8.0597	9.3434	14.05	0.362
	α-Fe_2_O_3_	47.99	Trigonal,R-3c:H	5.0369	5.0369	13.7552		
5	FeVO_4_	76.48	Triclinic, P-1	6.7139	8.0569	9.3489	14.90	0.949
	α-Fe_2_O_3_	23.52	Trigonal,R-3c:H	5.0366	5.0366	13.7545		
10	FeVO_4_	90.29	Triclinic,P-1	6.7111	8.0502	9.3387	14.87	1.258
	α-Fe_2_O_3_	9.71	Trigonal,R-3c:H	5.0301	5.0301	13.7550		
15	FeVO_4_	92.84	Triclinic,P-1	6.7140	8.0562	9.3458	13.67	1.268
	α-Fe_2_O_3_	7.16	Trigonal,R-3c:H	5.0358	5.0358	13.7444		
