# Supplementary material for: Fast interfacial charge transfer in α-Fe2O3−δCδ/FeVO4−x+δCx−δ@C bulk heterojunctions with controllable phase content
Source: Sci Rep. 2016 Dec 7;6:38603. doi: 10.1038/srep38603 (PMC5141511; doi:10.1038/srep38603)
Supplement: Supplementary Information [file srep38603-s1.doc]

Fast interfacial charge transfer in α-Fe_2_O_3-δ_C_δ_/FeVO_4-x+δ_C_x-δ_@C bulk heterojunctions with controllable phase content

Chengcheng Zhao, Guoqiang Tan*, Wei Yang, Chi Xu, Ting Liu, Yuning Su, Huijun Ren, and Ao Xia

*School of Materials Science and Engineering,* *Shaanxi University of Science & Technology, Xi’an 710021, China*

*Email address: tan3114@163.com (G.Q. Tan)*


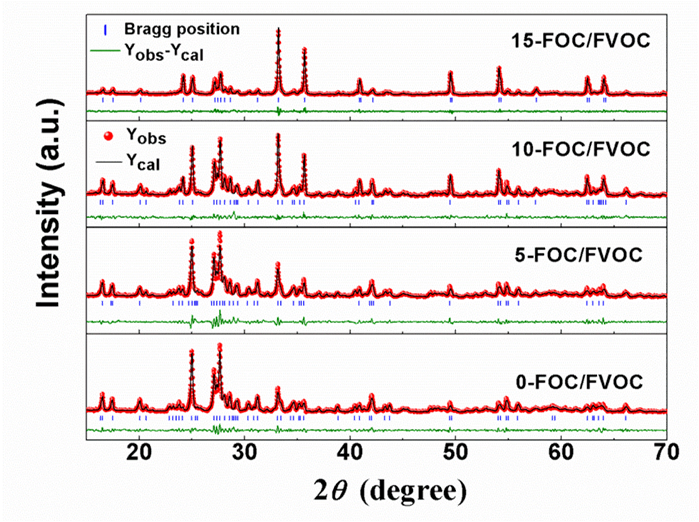


Fig. S1 Rietveld refined XRD patterns of FOC/FVOC heterojunctions


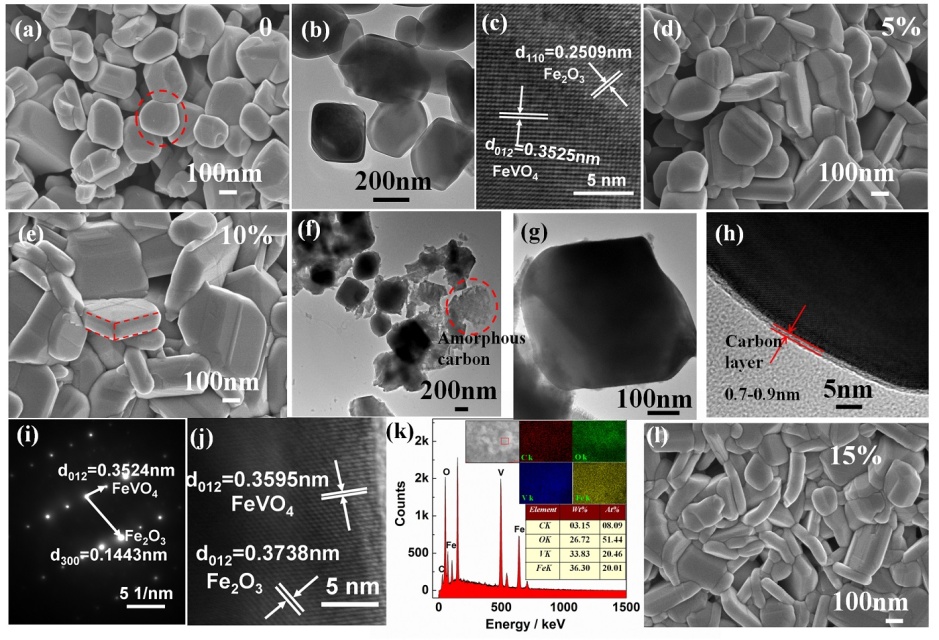
 Fig. S2 The SEM, TEM, HRTEM images of (a-c) 0-FOC/FVOC, (d) 5-FOC/FVOC, (e-j) 10%-FOC/FVOC and (i) 15-FOC/FVOC; (k) EDS spectrum of 10-FOC/FVOC


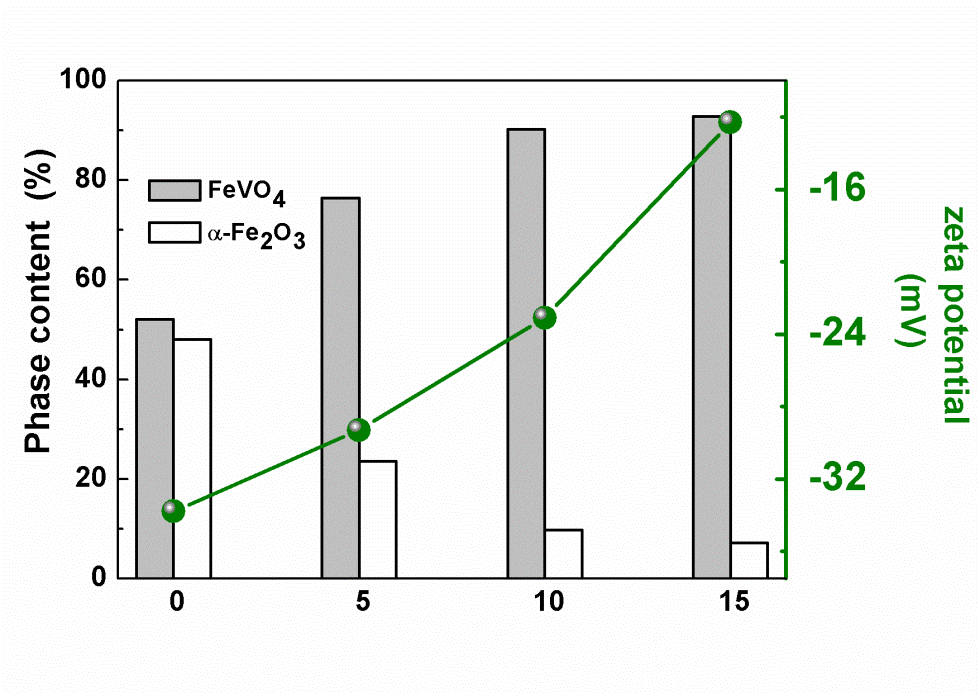


Fig. S3 Compare of the final phase contents and their pH values and zeta potentials (ζ values)

Table S1. Z-fit Equivalent Circuit Data of FOC/FVOC Films

| CDC code | R(Ω) | Q  (×10^-5^ Ssec^n^) | n | R_1_(Ω) | C(μF) | R_2_(kΩ) | |
| --- | --- | --- | --- | --- | --- | --- | --- |
|  |  |  |  |  |  | R_2_ value | error coefficient % |
| 0-FOC/FVOC | 28.49 | 9.412 | 0.8437 | 12240 | 356.8 | 53.15 | 3.689 |
| 5-FOC/FVOC | 38.54 | 11.46 | 0.8238 | 30.21 | 9.55 | 16.16 | 1.111 |
| 10-FOC/FVOC | 28.60 | 14.65 | 0.5676 | 47.25 | 57.61 | 8.29 | 2.924 |
| 15-FOC/FVOC | 23.67 | 10.95 | 0.6462 | 15.29 | 45.75 | 19.05 | 3.343 |
